# The multifaceted landscape of T cell exhaustion in organ transplantation: From molecular mechanisms (epigenetics, transcription, metabolism) to induction strategies

**DOI:** 10.1016/j.gendis.2025.101965

**Published:** 2025-12-06

**Authors:** Yining Wang, You Wu, Yufei Shen, Yujia Chen, Yifan Zhao, Xiandong Zeng, Kang He

**Affiliations:** a Department of Liver Surgery and Liver Transplantation, Renji Hospital, School of Medicine, Shanghai Jiao Tong University, Shanghai 200127, China; b School of Medicine, Shanghai Jiao Tong University, Shanghai 200127, China; c Shanghai Jiao Tong University, Shanghai 200127, China

**Keywords:** Epigenetic remodeling, Metabolic reprogramming, T cell exhaustion, Transplantation, Immunotherapy

## Abstract

T cell exhaustion is a state of T cell dysfunction resulting from persistent antigenic stimulation, characterized primarily by the high expression of inhibitory receptors, metabolic reprogramming, and epigenetic remodeling. T cell exhaustion is closely associated with immune responses in chronic infections, tumor escape, and organ transplantation. In transplantation immunology, T cell exhaustion plays a dual role: moderate exhaustion can promote immune tolerance and reduce graft rejection, while excessive exhaustion may weaken the defensive capabilities of the immune system, increasing the risk of infection and tumorigenesis. Therefore, effective regulation of T-cell exhaustion has become a crucial issue in the field of immunotherapy. Epigenetic or metabolic interventions may offer novel insights for achieving graft-specific tolerance. Further studies can focus on precise modulation of T-cell exhaustion through metabolic reprogramming, epigenetic regulation, and immune checkpoint inhibition, ultimately enhancing the efficacy of transplantation immunology and immunotherapy. This review focuses on the molecular phenotype, metabolic patterns, and mechanisms of epigenetic changes in exhausted T cells. It also explores the research progress of T cell exhaustion in organ transplantation. Furthermore, the review introduces strategies to induce T cell exhaustion, discussing how these strategies can effectively reduce the side effects of immunosuppressive therapy and promote graft tolerance.

## Background

T cells, as crucial components of specific immune responses, play a significant role in various immune reactions, such as clearing pathogens, resisting tumors, and transplant rejection. T cells also exhibit high heterogeneity and can be categorized into multiple subsets based on distinct classification criteria. According to their activation status, T cells are broadly divided into naïve T cells, effector T cells, and memory T cells. Naïve T cells are mature T cells that have not encountered antigen stimulation. They reside in the G_0_ phase and are primarily responsible for antigen recognition. Upon receiving peptide-major histocompatibility complex (pMHC) stimulation presented by dendritic cells in peripheral lymphoid organs, they become activated and differentiate into effector and/or memory T cells. Effector T cells are the primary executors of immune responses. They emerge following T cell activation, proliferation, and differentiation in response to antigenic challenge. Based on cluster of differentiation (CD) marker expression, they are classified into CD4^+^ T cells and CD8^+^ T cells (cytotoxic T cells).

CD4^+^ T cells, which include T helper subsets such as Th1, Th2, Th17, and regulatory T cells (Tregs), recognize peptide–MHC complexes and modulate the function of diverse immune cells, including CD8^+^ T cells and dendritic cells, playing a complex and critical role in the immune system. Evidence suggests that during chronic viral infections and in tumor contexts, CD4^+^ T cells can undergo exhaustion similar to CD8^+^ T cells.^1^ Exhausted CD4^+^ T cells exhibit molecular phenotypes resembling those of exhausted CD8^+^ T cells, including up-regulated expression of inhibitory receptors such as programmed death-1 (PD-1), cytotoxic T lymphocyte antigen-4 (CTLA-4), and lymphocyte activation gene 3 (LAG-3).^2^ Additionally, CD39 is markedly increased on exhausted CD4^+^ T cells and serves as a distinctive surface marker for their exhaustion.^3^ Key transcription factors critical in CD8^+^ T cell exhaustion, such as thymocyte selection-associated high mobility group box factor (TOX) and Krüppel-like factor 4 (KLF4), are also up-regulated in exhausted CD4^+^ T cells.^4^ Furthermore, unique features like up-regulation of PR/SET domain 1 (PRDM1) and down-regulation of Th-inducing POZ-Kruppel factor (ThPOK) have been observed in exhausted CD4^+^ T cells and may contribute to the development of the exhausted state. However, due to the limited number of studies focusing on CD4^+^ T cell exhaustion, the precise mechanistic roles of transcription factors such as TOX remain unclear.^2^

CD8^+^ T cells, upon activation, differentiate into cytotoxic T lymphocytes, which mediate the specific killing of target cells. Memory T cells are derived from either effector T cells or naïve T cells. They participate in lymphocyte recirculation, exhibit long-term persistence (often for years), and rapidly differentiate into effector T cells upon re-exposure to the same antigen, thereby mediating secondary immune responses.^5^

T cell exhaustion represents a state of dysfunctionality that often occurs when the body is stimulated by certain persistent antigens (*e.g.*, viruses, bacteria, or tumor cells) for an extended period or during chronic infections.^6^ Under these conditions, T cells may gradually lose their original effector functions.

After studying normal and CD4 knockout mice infected with lymphocytic choriomeningitis virus (LCMV), researchers first proposed the concept of T cell exhaustion.^7^^,^^8^ Since then, researchers have repeatedly observed T cell exhaustion in human and animal infection models, such as in human chronic infections including acquired immunodeficiency syndrome (caused by human immunodeficiency virus, HIV), hepatitis B (caused by hepatitis B virus, HBV), and hepatitis C (caused by hepatitis C virus, HCV).^9^^,^^10^ Additionally, T cell exhaustion has also been linked to tumorigenesis^11^ and transplant rejection.^12^

**Figure 1:**
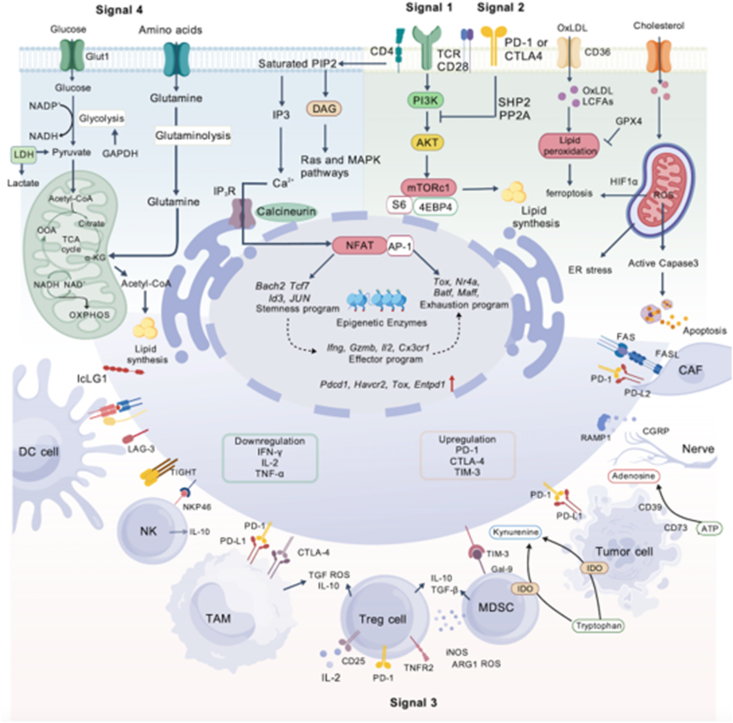
Overview of the changes in T cell exhaustion, including epigenetic regulation, transcription factors, and metabolic change. We reviewed the molecular phenotypic hallmarks of T cell exhaustion, focusing on the signaling pathways and downstream effects of key inhibitory receptors, including CTLA-4, PD-1, and Tim-3. The epigenetic reprogramming that occurs during exhaustion was delineated. We also highlighted the interplay among transcription factors such as TOX, NFAT, and MYB, and their roles in regulating cytokine production and cellular activation. Additionally, we examined the contributions of regulatory T cells (Tregs), macrophages, natural killer (NK) cells, and myeloid-derived suppressor cells (MDSCs) to the exhaustion process, and discussed the critical involvement of cytokines in this context. Finally, we reviewed the metabolic changes in T cell exhaustion, with more significant changes in metabolic pathways in glucose metabolism, lipid metabolism, and amino acid metabolism, which ultimately led to altered functions in T cells.

During the investigation of exhausted T cells, it has been observed that these cells exhibit up-regulated checkpoint molecules compared with normal T cells,^11^ such as the overexpression of inhibitory receptors (IRs).^13^ Additionally, exhausted T cells undergo physiological changes in both epigenetics and metabolic patterns (Fig. 1).^14^ In recent years, T cell exhaustion has been widely discussed in transplantation immunology, with researchers proposing it as a novel therapeutic approach for organ transplantation.^12^ Appropriate levels of T cell exhaustion can suppress excessive immune responses, thereby enhancing graft tolerance. In clinical settings, harnessing these regulatory mechanisms to precisely induce T cell exhaustion and promote graft tolerance without compromising immune surveillance represents a significant breakthrough for future transplantation immunology research and treatment.

## Molecular phenotypes of T cell exhaustion

**Figure 2:**
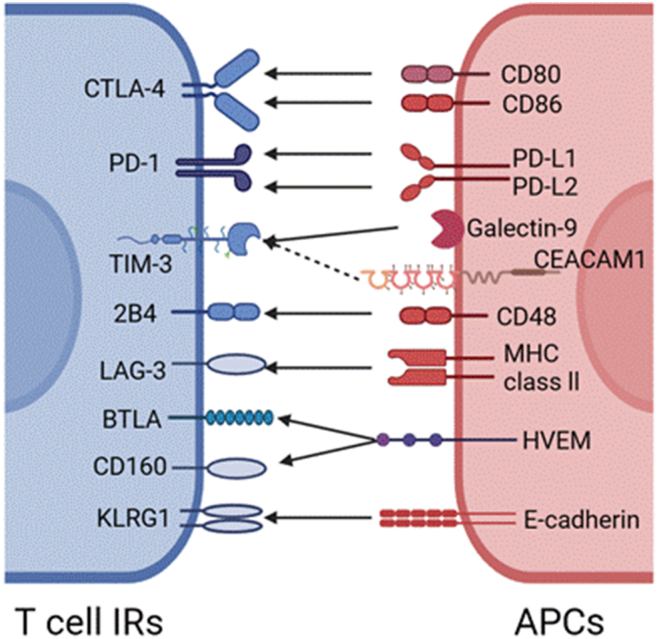
Common molecular phenotypes of T cells.

Exhausted T cells represent a specific differentiation state of T cells during the infection process, characterized by distinct molecular phenotypes (Fig. 2). Researchers have discovered that exhausted T cells abundantly express IRs, such as CTLA-4, PD-1, T cell immunoglobulin and mucin domain-containing protein 3 (TIM-3), LAG-3, 2B4 (CD244), B and T lymphocyte attenuator (BTLA), CD160, and killer-cell lectin like receptor G1 (KLRG1). In the following sections, we will provide detailed descriptions of four typical molecular phenotypes: CTLA-4, PD-1, TIM-3, and LAG-3.

## CTLA-4

CTLA-4, also known as CD152, stands as a pivotal immune checkpoint molecule recognized as a negative regulator of T cell priming.^15^ CTLA-4 binds to B7 family molecules (B7-1/CD80 and B7-2/CD86) with significantly higher affinity than the costimulatory molecule CD28, thereby competitively inhibiting CD28-mediated costimulatory signals. Through its intracellular domain, CTLA-4 recruits phosphatases such as Src homology-containing protein 2 (SHP-2) and protein phosphatase 2A (PP2A), suppressing key molecules in the T cell receptor (TCR) signaling pathway (*e.g.*, phosphoinositide-3-kinase (PI3K)-protein kinase B (AKT) and mitogen-activated protein kinase (MAPK) pathways), which consequently inhibits T cell proliferation and cytokine production. In chronic infections and tumor microenvironments, the elevated expression of CTLA-4 is closely associated with T cell exhaustion. Specifically, CTLA-4 promotes T cell dysfunction and exhaustion by inhibiting T cell activation and proliferation. Furthermore, CTLA-4 is highly expressed in Tregs, indirectly exacerbating the progression of T cell exhaustion by suppressing the activation of effector T cells and promoting immune resistance.^16^^,^^17^

## PD-1

PD-1 is a profound immunosuppressive receptor that is highly expressed on exhausted T cells. Upon binding to its ligands, programmed death ligand 1 (PD-L1) and PD-L2, PD-1 recruits the phosphatase SHP-2 through its intracellular domain. This recruitment leads to the inhibition of key molecules in the TCR signaling pathway, such as zeta chain of T cell receptor-associated protein kinase 70 (ZAP70) and PI3K, thereby suppressing the priming, proliferation, and effector functions of T cells. In chronic infection and tumor environments, the persistent expression of PD-1 results in T cell exhaustion, manifesting as reduced cytokine production and decreased proliferative capacity. PD-1 synergizes with CTLA-4 to further exacerbate the functional suppression of T cells. Notably, PD-1 primarily acts during the effector phase of T cells, while CTLA-4 exerts its inhibitory effects during the early stages of T cell priming.^18^^,^^19^

## Tim-3

Tim-3 serves as a crucial immunomodulatory molecule, often regarded as a negative regulator of T-cell priming. Its ligand, Galectin-9, binds to the sugar chains on Tim-3 through its carbohydrate recognition domain (CRD), inducing calcium influx in Th1 cells. This activation triggers the calpain and caspase pathways, ultimately leading to the apoptosis of Th1 cells.^20^, ^21^, ^22^ In chronic inflammatory conditions and the tumor microenvironment, Tim-3 is co-expressed with PD-1 on exhausted CD8^+^ T cells. The combined blockade of both PD-1/PD-L1 and Tim-3/Galectin-9 pathways can restore the functionality of CD8^+^ T cells and ameliorate the disease state.^21^ The mechanism of action of Tim-3 is multifaceted. On one hand, it may operate similarly to PD-1,^23^ inhibiting immune synapse stability and Lck phosphorylation, thereby blocking TCR signaling and suppressing T-cell priming.^24^ On the other hand, Tim-3 might also exert a co-stimulatory effect, facilitating TCR signaling, maintaining T-cell function, delaying exhaustion, or exerting a “rescue” role.^23^^,^^25^

## LAG-3

LAG-3 is an inhibitory receptor that is highly expressed on exhausted T cells.^26^ LAG-3 works synergistically with PD-1 to promote the expression of TOX, initiating the cellular exhaustion program.^27^, ^28^, ^29^ In this process, LAG-3 plays a “striking” leading role.^27^ The simultaneous binding of LAG-3/TCR and its ligands inhibits TCR:CD3-dependent intracellular calcium flux, thereby suppressing downstream signal transduction. Indeed, following the binding of TCR to its ligands in CD8 and CD4 T cells, LAG-3 can also physically associate with TCR, down-regulating TCR-dependent signaling cascades and thus inhibiting T cell effector functions.^30^

## Differences in molecular phenotypes across organs

The variation in exhaustion markers across different transplanted organs may be attributed to organ-specific immune microenvironments, post-transplant immunotherapy, and associated complications. In kidney transplantation, interactions between conventional immunosuppressive drugs and cancer immune checkpoint inhibitors can modulate the expression of markers such as PD-1, LAG-3, and CTLA-4. Compared with organs such as the liver and lungs, kidney transplants exhibit a higher propensity for rejection.^31^ In hematopoietic stem cell transplantation, complications such as Epstein–Barr virus (EBV) infection have been shown to influence the expression profiles of T cell exhaustion markers. Studies indicate that patients with EBV reactivation exhibit elevated levels of TIM-3 on T cells (including both CD8^+^ and CD4^+^ subsets) in the early post-transplant phase, which is associated with delayed immune reconstitution and impaired cytotoxic function.^32^

## T cell exhaustion and epigenetic regulation

### Epigenetic modifications associated with T cell exhaustion

The mechanisms of epigenetics mainly include DNA methylation and post-translational modifications of histones, which influence gene expression without altering the DNA nucleotide sequence.^33^ DNA methylation involves the addition of a methyl group to the 5′ position of the cytosine ring in cytosine-phosphate-guanine (CpG) dinucleotides. These dinucleotides are often located in regions known as CpG islands, which are associated with gene promoters or enhancers. Histone modifications alter the structure of nucleosomes through processes such as acetylation, methylation, phosphorylation, and ubiquitination, thereby affecting gene accessibility and expression. Typically, increased gene accessibility is associated with acetylation mediated by histone acetyltransferases, while decreased gene activity is related to deacetylation mediated by histone deacetylases.^34^ These molecular features are strictly regulated by environmental stimuli and can be inherited during meiosis and mitosis, playing a crucial role in the development and function of the immune system.^35^

Early epigenetic analyses of naive CD8^+^ T cells have shown that DNA methylation, histone modifications, and chromatin accessibility play key regulatory roles during activation, effector responses, and memory formation of T cells in response to viral infections.^36^^,^^37^ T cell exhaustion is associated with widespread transcriptomic and epigenetic remodeling, resulting in distinct chromatin profiles and differentiation programs compared with effector and memory T cells.^19^^,^^38^^,^^39^ Multiple studies have identified open chromatin sites linked to T cell exhaustion in LCMV-specific and tumor-specific CD8^+^ T cells.^40^^,^^41^ Comparative analyses indicate that exhausted T cells undergo genome-wide epigenetic reprogramming, generating thousands of differentially accessible regions. Many of these regions are adjacent to genes that mediate the differentiation of exhausted T cells, such as interferon-gamma (*Ifng*, encoding IFN-γ) and programmed cell death protein 1 (*Pdcd1*, encoding PD-1). These findings suggest that exhausted T cells possess a unique chromatin state, characterized by distinct epigenetic features, which may be targeted for therapeutic intervention.

A study performed single-cell transposase-accessible chromatin sequencing (scATAC-seq) and single-cell multi-omics sequencing (scRNA-seq + scATAC-seq) on CD8^+^ T cells from various tissues and organs, revealing distinct epigenetic landscapes across different organs.^42^ For example, the chromatin accessibility of genes associated with memory T cell formation, such as the motifs of FOSB, FOS, FOSL1, and BACH transcriptional regulator 2 (BACH2), was significantly lower in the liver compared with organs like the skin. In contrast, chromatin accessibility was higher for genes involved in interferon signaling pathways. These findings suggest that the liver may be less permissive to the development of immunological memory relative to other organs, a phenomenon potentially linked to the persistent stimulatory environment created by the liver’s portal venous blood supply.

Currently, epigenetic interventions in transplantation are primarily aimed at controlling disease recurrence and post-transplant complications. Several related clinical trials are either ongoing or have been completed. For example, in patients with high-risk acute myeloid leukemia following allogeneic hematopoietic stem cell transplantation, the use of two epigenetic modulators, panobinostat and decitabine, has been shown to improve survival and suppress relapse.^43^ However, no clinical trials have yet been conducted to explore the application of epigenetic interventions in modulating T cell exhaustion or establishing immune tolerance.

### Epigenetic reprogramming and its stability

DNMT3A, an enzyme known as a *de novo* DNA methyltransferase, plays a pivotal role in regulating the epigenetic reprogramming of T cells as they transition from an effector to an exhausted state during chronic infections such as LCMV. It has been established that T cells lacking or with knocked-out DNMT3A exhibit significantly reduced epigenetic silencing at multiple key gene loci, including Ifnγ, Tcf7, Ccr7, and Tbx21.^44^^,^^45^ These cells maintain their effector functions and exhibit reduced exhaustion upon repeated antigenic stimulation, highlighting the critical involvement of DNMT3A in the process of T cell exhaustion. On the other hand, suppressor of variegation 3–9 homolog 1 (SUV39H1), a histone methyltransferase responsible for the trimethylation of histone H3 at lysine 9, is typically associated with transcription and silencing of heterochromatin. Studies in the context of chimeric antigen receptor-T cell therapy have revealed that chimeric antigen receptor-T cells lacking SUV39H1 demonstrate significant advantages in terms of expansion, persistence, and anti-tumor efficacy.^46^ These findings suggest that SUV39H1 may regulate T cell exhaustion by altering histone methylation patterns, thereby influencing the accessibility of genes related to T cell effector functions and memory formation.

In T cell exhaustion, there exists a significant interplay between cellular metabolism and epigenetic regulation. Exhausted T cells undergo metabolic reprogramming, characterized by suppressed glycolysis and impaired metabolism of certain amino acids such as leucine. These aspects will be elaborated in Section 5. Metabolic changes in T cell exhaustion. Acetyl-CoA occupies a central position in cellular metabolism and serves as the key donor for histone acetylation—a critical epigenetic modification. Metabolic reprogramming can modulate intracellular levels of acetyl-CoA, thereby influencing histone acetylation status and subsequently regulating gene expression.^47^ Similarly, histone methylation can be modulated by metabolic processes through its methyl donor, S-adenosylmethionine (SAM).^48^ Additionally, emerging evidence suggests that epigenetic modifications may exert feedback regulation on metabolic pathways.^49^

Adenosine monophosphate-activated protein kinase (AMPK) serves as a central metabolic sensor and a master regulator of cellular energy homeostasis and mitochondrial integrity.^50^ It contributes to chromatin remodeling through histone phosphorylation, acetylation, and demethylation, thereby enhancing the expression of genes that promote cell survival. Furthermore, AMPK modulates other epigenetic regulators, for instance, by activating serine hydroxymethyltransferase-2 (SHMT2), which ultimately enhances the activity of DNMT3a and DNMT3b, thereby exerting broad epigenetic influence.^51^ Studies have demonstrated that AMPK deficiency leads to a reduction in effector T cells (both CD4^+^ and CD8^+^) and an increase in the proportion of Tregs.^52^ In the context of transplantation, there is significant clinical interest in strategically modulating AMPK activity to attenuate T cell-mediated immune rejection, while minimizing detrimental effects on cellular metabolism and mitochondrial homeostasis.

It is noteworthy that T cell exhaustion exhibits epigenetic stability.^19^^,^^38^^,^^53^ Continuous antigenic stimulation induces epigenetic reprogramming, ultimately leading to the establishment of irreversible epigenetic scarring. Once T cell exhaustion occurs, even in the absence of antigen exposure, T cells remain in an “exhausted” state characterized by reduced effector function, cytotoxicity, and proliferative capacity.^53^ Therefore, exploiting the plasticity and stability of epigenetics to induce or circumvent T cell exhaustion holds considerable potential in immunotherapy.^44^

## T cell exhaustion and transcription factors

### Transcription factors associated with T-cell exhaustion

TOX has been identified as a crucial transcription factor in the process of T-cell exhaustion, selectively up-regulated during the development of exhausted T cells and playing a significant role in this phenomenon.^44^^,^^54^ In the early stages of chronic infection, TCR signaling induces calcium flux, which triggers the expression of TOX. TOX is capable of inducing the expression of numerous key individual exhaustion genes, such as inhibitory receptors (Pdcd1, Lag3, Ctla4) and transcription factors (Nr4a2, Ikzf3, Tox2, Bhlhe41). Furthermore, TOX can alter the epigenetic landscape of cells, modifying transcription and chromosomal accessibility, and suppressing the generation of effector and memory cells, ultimately leading to irreversible T-cell exhaustion.^19^^,^^38^^,^^44^^,^^54^ The role of TOX is closely linked to nuclear receptor 4A (NR4A), another transcription factor involved in T-cell exhaustion, and there is clear evidence indicating that these two factors form a positive feedback loop.^55^

Nuclear factor of activated T cells (NFAT) is a transcription factor with dual roles, functioning in both T cell activation and exhaustion.^56^ On one hand, NFAT proteins interact with activator protein-1 (AP-1), forming a cooperative NFAT:AP-1 complex that induces the expression of cytokine genes and other activation-related genes.^57^ On the other hand, “unpartnered” NFAT, which does not bind to AP-1, controls CD8^+^ T cell exhaustion by directly binding to regulatory regions of many exhaustion-related genes, including the promoters and cis-regulatory regions of Pdcd1 (PD-1) and hepatitis A virus cellular receptor 2 (Havcr2 or TIM3). Moreover, it has been demonstrated that there is a distinct positive feedback loop between NFAT, TOX, and NR4A family transcription factors during T cell exhaustion.^55^ NFAT, interferon regulatory factor 4 (IRF4), and basic leucine zipper transcription factor ATF-like (BATF) constitute a critical axis associated with T cell exhaustion. The positive feedback loop of NFAT drives the expression and interaction of transcription factors IRF4 and BATF, promoting the differentiation process of exhausted T cells.^44^^,^^57^^,^^58^

MYB is crucial for the formation of progenitor exhausted T cells (CD62L^+^ TPEX).^44^^,^^59^ Progenitor exhausted T cells are a group of stem-like cells capable of self-renewal and differentiation into exhausted T cells. MYB maintains the exhausted state of T cells and their long-term self-renewal ability by regulating the expression of a range of genes. These include molecules related to lymph node homing (such as CD62L, CCR7, S1PR1), cell cycle inhibitors (*e.g.*, CDKN1B, CDKN2D), and quiescence factors (*e.g.*, KLF2, KLF3). This dual role ensures that during chronic infections, T cells can persist and maintain a certain immune response while avoiding excessive immune damage.^59^ Additionally, MYB promotes the transcription of transcription factor-1 (TCF-1), a key transcription factor in exhausted T cell progenitors (TexProg) encoded by the Tcf-7 gene, which helps maintain T cell quiescence.^60^ When activated under the influence of MYB, NFAT, and BATF, TCF-1 can collaborate with BACH2 to promote progenitor cell differentiation. In the early stages of chronic infection, it suppresses the differentiation of effector T cells and promotes the generation of exhausted T cell populations.^44^^,^^61^^,^^62^

By applying ATAC-seq and RNA-seq technologies, it was observed that significant alterations in chromatin accessibility occurred at the binding sites of specific transcription factors, including E2A, BATF, IRF4, T-bet, and TCF1, within exhausted T cells. These transcription factors are implicated in regulating the development and function of exhausted T cell subsets.^41^ BATF and IRF4 represent a pair of emerging regulatory factors that modulate immune cell development and function through their interaction. IRF4, particularly, plays a key role in regulating the differentiation of Th cells and effector T cells.^63^ RNA sequencing analysis of Irf4^−/−^ CD4^+^ T cells revealed a reduction in the expression of genes associated with effector T helper cells and an increase in the expression of genes related to Tregs. These findings suggest that IRF4 deficiency alters T cell differentiation trajectories, promoting Treg formation—a shift that may contribute to the development of T cell exhaustion. Furthermore, in a mouse model of hematopoietic stem cell transplantation, recipients of Irf4^−/−^ CD4^+^ T cells exhibited attenuated graft-versus-host disease (GVHD)-related symptoms and reduced colitis. This indicates that the BATF–IRF4 axis may play a pivotal role in post-transplant immune reconstitution and complication pathogenesis by modulating T cell differentiation and function.^64^

### The temporal characteristics of transcription factor regulation in T cell exhaustion

Through ATAC-seq analysis of T cell subsets in LCMV infection, researchers have identified stage-specific transcription factor activity patterns in exhausted T cells. As summarized by Belk et al,^38^ progenitor exhausted T cells show increased TCF-1 and BACH2 activity; transitory exhausted T cells exhibit enriched binding sites for T-bet and RUNX factors; and terminal exhausted T cells demonstrate enrichment of NR4A and EOMES binding sites. Furthermore, Martínez et al confirmed NFAT’s role in T cell exhaustion by engineering CA-RIT-NFAT1, an AP-1 interaction-deficient mutant, revealing its AP-1-independent contribution to exhaustion.^56^

Based on the findings,^38^ we have delineated the temporal dynamics of transcription factor regulation during T cell exhaustion: Initially, NFAT proteins modulate the balance between productive T cell activation and T cell dysfunction downstream of TCR signaling.^56^ The NFAT:AP-1 heterodimer promotes T cell activation, while the “unpartnered” NFAT directly binds and induces the expression of exhaustion-related genes and TOX.^55^ Exhausted T cell progenitors maintain the expression of Tcf7 (encoding TCF-1), which may be initially driven by BACH2, enabling their self-renewal and proliferation.^65^ Following persistent antigenic stimulation, T cells enter a transient “exhausted” state, yet retain partial effector activity driven by T-bet. Finally, T cells differentiate into terminally exhausted T cells, where the sustained expression of TOX induces the up-regulation of transcription factors such as EOMES and NR4A. These factors, in turn, contribute to T cell exhaustion, manifesting as alterations in the epigenetic landscape, increased expression of inhibitory receptors, and diminished proliferative and effector functions.^19^^,^^38^^,^^44^^,^^54^^,^^55^

## Metabolic changes in T cell exhaustion

### Metabolic reprogramming associated with T cell exhaustion

Lymphocytes are highly adaptable to environmental changes.^66^ Upon TCR stimulation, Naïve T cells increase their metabolic rate by enhancing substrate absorption (glycolysis, amino acid metabolism, lipid metabolism, *etc*.), catabolism, and nutrient synthesis.^67^ Comparatively, exhausted T cells show unique metabolic characteristics. Researchers have found that exhausted T cells can be classified according to phenotypic and functional differences^68^: Progenitor exhausted T cells are metabolically characterized by mitochondrial fatty acid oxidation and oxidative phosphorylation, while terminally exhausted T cells rely mainly on impaired glycolytic metabolism.^69^, ^70^, ^71^

Within the transplant microenvironment, progenitor and terminally exhausted T cells exhibit significant differences in energy sources and metabolic substrate utilization. Progenitor exhausted T cells retain relatively higher mitochondrial function and can up-regulate fatty acid oxidation (FAO)-related genes (*e.g.*, Cpt1a) under conditions of limited glucose availability or impaired glycolysis, thereby sustaining basal energy production via mitochondrial oxidative phosphorylation. Although their spare respiratory capacity is lower than that of acute effector T cells, it remains higher than that of terminally exhausted subsets, indicating preserved metabolic plasticity. In contrast, terminally exhausted T cells demonstrate severely compromised glycolytic capacity, characterized by down-regulation of key glycolytic enzymes and transporters such as glucose transporter protein type 1 (GLUT1), hexokinase 2 (HK2), and glyceraldehyde-3-phosphate dehydrogenase (GAPDH), resulting in reduced glucose uptake, diminished lactate production, and inefficient ATP generation.^71^ Furthermore, these cells frequently exhibit mitochondrial dysfunction, manifested as reduced mitochondrial membrane potential, enlarged mitochondrial volume, and aberrant morphology. They also display heightened dependence on glucose supply and an inability to transition to oxidative phosphorylation under low-glucose conditions. Functional T cells (*e.g.*, CMV-specific T cells) can maintain effector functions via oxidative phosphorylation in the absence of glucose, whereas exhausted HBV-specific T cells lack this adaptive metabolic flexibility.^72^ The T cells mentioned below generally refer to terminally exhausted T cells.

**Figure 3:**
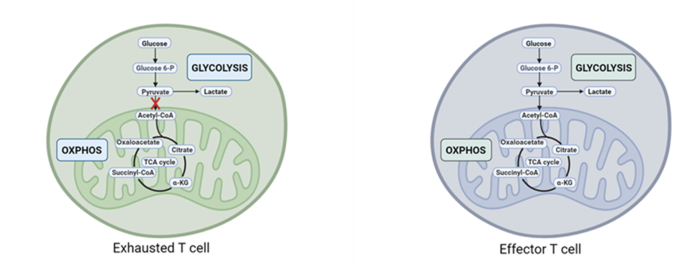
Alterations in glucose metabolism across different T cell subsets. Exhausted T cells exhibit reduced glucose uptake and decreased glycolysis levels, while effector T cells rely on glycolysis for rapid energy supply to support their proliferation, differentiation, and effector functions.

Aerobic glycolysis can generate key metabolic intermediates, which supply energy and promote the proliferation of activated T cells.^73^ Normally, upon activation, effector T cells rapidly increase glucose uptake and metabolism through glycolysis.^67^ Glucose metabolism in exhausted T cells shows significant abnormalities when compared with normal effector T cells. For example, expression of glucose transporters (GLUT1) and glycolysis-related enzymes (GAPDH) decreases in exhausted T cells,^71^^,^^74^ resulting in lower glucose uptake and suppressed glycolysis, which leads to insufficient energy supply for T cells and limitation of immediate immune response.^71^ Due to this metabolic change, exhausted T cells no longer rely on their original metabolic mode for energy synthesis. It results in an inability to effectively maintain high levels of ATP synthesis and suppresses cell proliferation and function (Fig. 3).

Reprogramming in amino acid metabolism, such as changes in glutamine, tryptophan, and leucine metabolism, also plays an important role in T cell activation and function maintenance.^67^ Glutamine promotes acetylation or lipid synthesis in T cells and enhances the transport of other amino acids, while in exhausted T cells, they show lower ability to transport and utilize amino acids, leading to insufficient energy and limited function.^67^^,^^75^ The products of tryptophan metabolism (such as kynurenine) through the indoleamine-2,3-dioxygenase (IDO) pathway inhibit T cell function, especially exacerbating T cell exhaustion in the tumor microenvironment.^67^^,^^76^ In addition, changes in leucine metabolism are crucial. High levels of leucine and arginine promote T cell proliferation and function by activating the mechanistic target of rapamycin complex 1 (mTORC1) signaling pathway.^18^ Generally, leucine maintains mTORC1 activity. However, if exhausted, its metabolism would be inhibited, resulting in lower mTORC1 activity, thereby leading to a similar inhibitory effect. For instance, mTOR inhibitors such as everolimus and sirolimus are shown to inhibit tumor growth and cell proliferation in hepatocellular carcinoma animal models.^77^ Therefore, sirolimus (SRL) is a commonly used immunosuppressant in clinical use, and is often used in patients who are ready to undergo liver transplantation for hepatocellular carcinoma.^78^

**Figure 4:**
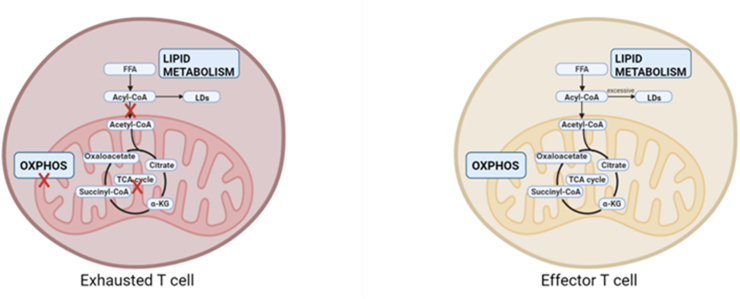
Variations in lipid metabolism among distinct T cell subpopulations. Exhausted T cells switch their energy pathway to fatty acid oxidation, whereas effector T cells enhance their fatty acid synthesis capabilities when glucose metabolism is limited.

Alteration in lipid metabolism is another important feature. FAO is an essential pathway for maintaining mitochondrial function and ATP synthesis to support T cell proliferation and effector function.^79^ When glycolysis is inhibited, effector T cells will switch from catabolism to anabolism, which means they will increase fatty acid synthesis (FAS) to provide long-term energy support.^67^ On the contrary, exhausted T cells in the early stage turn energy pathways upside down. They switch energy metabolism from FAS to FAO.^68^ In addition, TCR-mediated AKT/PI3K/mTORC1 signaling up-regulates lipogenic enzyme transcription through sterol regulatory element binding protein (SREBP) to promote the synthesis of cholesterol and fatty acid.^80^ The accumulation of cholesterol exacerbates endoplasmic reticulum stress, thereby promoting the expression of co-inhibitory receptors such as PD-1, and ultimately inducing exhaustion of CD8^+^ T cells (Fig. 4). Studies have demonstrated that cholesterol metabolism plays a critical role in driving or regulating T cell exhaustion. Specifically, abnormal intracellular cholesterol accumulation in CD8^+^ T cells induces endoplasmic reticulum stress, activates the X-box binding protein 1 (XBP1) signaling pathway, and up-regulates inhibitory receptors such as PD-1 and TIM-3, thereby reinforcing the exhausted phenotype.^81^ Complementarily, inhibiting cholesterol esterification (via ACAT1 suppression) increases membrane cholesterol availability, promotes immune synapse formation, and restores effector function in CD8^+^ T cells.^82^ These findings indicate that cholesterol metabolism is not merely a passive metabolic feature but an active regulator of the exhaustion pathway.

Furthermore, regarding whether metabolic reprogramming is universally present across various types of transplantation, current evidence indicates that although exhausted T cells consistently demonstrate reduced glycolytic capacity and enhanced dependence on FAO in multiple transplant settings,^71^ these findings are largely derived from chronic infection models or *in vitro* systems. Comparative studies across different solid organ transplants, such as liver, kidney, and heart, remain limited. In liver transplantation tolerance models, infiltrating CD8^+^ T cells have been observed to exhibit classical exhaustion markers (*e.g.*, high expression of PD-1, LAG3, TIM-3, TIGIT) and impaired effector function, yet their glycolytic or FAO activity has not been quantitatively assessed.^83^ Similarly, while exhausted T cells have been reported in kidney and heart transplant models, direct measurements of metabolic pathways, particularly comparative analyses of glycolysis and fatty acid oxidation, are still lacking.^84^ Thus, available data support the occurrence of a metabolic shift from glycolysis to FAO in T cell exhaustion cells, but do not yet confirm that this transition is universal or consistent in magnitude across all transplant types. In other words, organ-specific variations in metabolic behavior are likely, but direct comparative experimental evidence is urgently needed. Future studies should employ integrated transcriptomic, metabolomic, and metabolic flux analyses in liver, kidney, and heart transplant models to systematically validate these patterns.

### T cell exhaustion in organ transplantation

Organ transplantation is a remarkable achievement in modern medicine, serving as a critical intervention for numerous end-stage diseases. T cell exhaustion plays an important role in organ transplantation, while presenting opposite characteristics. It can either promote graft tolerance or, conversely, weaken the body’s immune defense due to excessive exhaustion.^12^^,^^85^^,^^86^ T cell exhaustion and its subsequent impact on transplantation outcomes vary depending on the specific organ involved (the liver, kidney, heart, *etc*.), influenced by factors like antigenic load.^85^^,^^87^ Conclusively, organs with a higher antigenic load (*e.g.*, the liver) are generally more prone to inducing T cell exhaustion and immune tolerance compared with smaller organs.^88^^,^^89^ Furthermore, the age of both the recipient and the donor influences the T cell rejection process following transplantation. For instance, organs from elderly donors may exhibit increased immunogenicity, thereby elevating the risk of rejection. Conversely, elderly recipients often experience a decline in T cell function due to immunosenescence and the accumulation of exhausted T cells, resulting in lower acute rejection reactions.^90^ Studies have demonstrated that in elderly donors/recipients, markers of T-cell exhaustion, such as PD-1 and LAG-3, are significantly up-regulated, while there is a concurrent marked decline in naïve T-cell subsets, hallmarks of immunosenescence.^91^ The following sections delve into the specific manifestations of T cell exhaustion in the context of different organ transplantations.

### Kidney transplantation

In kidney transplantation, T-cell exhaustion exhibits a complex role.^85^ Moderate T-cell exhaustion can contribute to graft tolerance by regulating the response of alloreactive T cells. It has been shown that the proportion of exhausted T cells positively correlates with renal function six months after transplantation, measured via estimated glomerular filtration rate,^84^ suggesting that moderate T-cell exhaustion may be associated with better graft function. However, excessive T-cell exhaustion can potentially weaken the body’s ability to control viral infections and tumors, thereby increasing the risk of transplantation failure.^85^

Furthermore, tissue-resident memory T cells may play a fundamental role in the process of chronic rejection at the transplantation site.^85^ Memory T cells serve as a crucial immune barrier in transplantation, which has a rapid antigen response. Although a subset of tissue-resident memory T cells undergoes exhaustion and promotes graft tolerance, the remaining active T cells may counteract this promotion, offsetting the beneficial effects of T-cell exhaustion on graft tolerance.

### Heart transplantation

Research on T-cell exhaustion in the context of heart transplantation has primarily focused on hearts that carry viruses. When the donor heart harbors LCMV, T-cell exhaustion occurs in the recipient mice, a process facilitated by the cytokine interleukin-10 (IL-10).^92^ In transplantation cases involving EB virus infection, individuals with high viral loads in their hearts exhibit distinct CD8^+^ T-cell characteristics, including the accumulation of terminally exhausted T cells.^93^ While T-cell exhaustion may favor graft survival, it complicates the clearance of viruses and increases the susceptibility to virus-related complications.^92^^,^^93^ Therefore, balancing T-cell exhaustion and the function of memory T cells is crucial for the success of heart transplantation.

## Liver transplantation^94^

The liver is an organ with a high antigen load in the human body. On one hand, the liver itself has a large volume; on the other hand, it receives blood from the gastrointestinal tract via the portal vein. This blood is rich in dietary antigens and various gut commensal microorganisms, representing a significant antigenic burden and creating a persistently stimulatory environment. Under chronic high antigen exposure, T cells undergo epigenetic reprogramming, up-regulation of certain transcription factors (*e.g.*, Blimp-1, TOX),^95^ mitochondrial dysfunction, and metabolic rewiring,^96^ all of which collectively promote exhaustion. Furthermore, the liver possesses a unique immune microenvironment. Kupffer cells in the liver highly express inhibitory ligands such as PD-L1, which engage with inhibitory receptors on T cells to suppress their function.^96^ Other immunosuppressive cells in the liver, including Tregs and myeloid-derived suppressor cells (MDSCs), can further exacerbate T cell exhaustion through direct contact or secretion of immunosuppressive factors like IL-10 and transforming growth factor-beta (TGF-β). Therefore, liver transplantation is more likely to induce T-cell exhaustion.^97^ Compared with transplants of other organs such as the heart or kidney, T cells after liver transplantation often exhibit massive activation and rapid proliferation, differentiating into effector or memory cells, followed by a transition into an exhausted (or unresponsive) state.^87^^,^^88^ Specifically, the proportion of PD-1^+^ CD8^+^ T cells in the peripheral blood of patients after liver transplantation increases dramatically, while the expression of cytokines (such as IFN-γ and IL-2) decreases considerably.^87^ Additionally, the acceptance of the graft in the presence of significant T-cell infiltration in the liver suggests that the T cells should be in an exhausted state. MDSCs play a crucial role in T-cell exhaustion after liver transplantation.^88^ The up-regulation of CD84 molecules on the surface of MDSCs activates downstream pathways, up-regulating the expression of PD-L1 and generating reactive oxygen species. This induces CD8^+^ T cells to express high levels of exhaustion markers (such as PD-1 and TIM-3), ultimately leading to T-cell exhaustion.

T-cell exhaustion can play a significant role in liver transplantation through various pathways, promoting graft survival and the success of transplantation. A study has shown that the high expression of inhibitory receptors on T-cell surfaces helps suppress excessive T-cell activation, thereby reducing ischemia-reperfusion injury after liver transplantation. Additionally, it maintains the homeostasis of the liver by reducing neutrophil and macrophage infiltration and promoting T-cell apoptosis.

### T cell exhaustion in hematopoietic stem cell transplantation

Hematopoietic stem cell transplantation is a common treatment for various hematological diseases, particularly significant in the treatment of malignant blood disorders such as leukemia and lymphoma. Enhancing graft-versus-leukemia (GVL) effects while minimizing GVHD remains a primary challenge in hematopoietic stem cell transplantation.^65^^,^^98^ The following discussion focuses on the role of T-cell exhaustion in the context of hematopoietic stem cell transplantation.

T-cell exhaustion significantly impacts the GVL effect. Donor T cells are the primary mediators of the GVL response, eliminating leukemia cells in the recipient and aiding in the prevention of leukemia relapse. However, certain highly heterogeneous diseases like acute myeloid leukemia may lead to T-cell exhaustion characterized by the up-regulation of inhibitory receptors (*e.g.*, PD-1, TIGIT, and TIM-3) and increased expression of exhaustion-associated transcription factors. For instance, PD-1 ligation down-regulates GVHD by modulating interferon-gamma (IFN-γ) production, ultimately elevating the risk of post-transplant leukemia relapse.^99^, ^100^, ^101^, ^102^ Consequently, it is noted that the alleviation of T-cell exhaustion is associated with effective anti-leukemia responses following reinduction therapy.^101^

Nevertheless, the GVL effect is intricately linked to the development of GVHD, and GVHD treatment can limit the GVL response.^103^ Senjo observed that donor T cells rapidly differentiate into exhausted T cells expressing PD-1 and T cell immunoreceptor with Ig and ITIM domains (TIGIT) after transplantation, with these T cells exhibiting severely impaired effector functions.^100^ In acute GVHD, the emergence of exhausted T cells is associated with persistent host antigen stimulation, and molecules like PD-1 and TOX play crucial roles in T-cell differentiation. The continuous expression of PD-1 attenuates donor T-cell aggressiveness towards the host by inhibiting T-cell proliferation and effector functions, potentially providing partial relief from GVHD. Conversely, T-cell exhaustion can also weaken anti-tumor immune surveillance, leading to a decreased GVL effect and an increased risk of relapse. Therefore, further research is needed to determine the appropriate therapeutic strategies for managing T-cell exhaustion in the context of hematopoietic stem cell transplantation.

### Induction of T cell exhaustion

T cell exhaustion, a specific state of T cell “dysfunction”, holds significant potential in immunotherapy, particularly in areas such as transplantation. Therefore, it is crucial to investigate methods that induce T cell exhaustion.

### Tregs

Tregs, specifically FOXP3^+^ CD4^+^ Tregs, play a complex and pivotal role in the process of T cell exhaustion.^104^ These cells are capable of inducing T cell exhaustion through multiple mechanisms.

Tregs can secrete various inhibitory cytokines, such as TGF-β and IL-10, which promote T cell exhaustion.^104^ In the tumor microenvironment, Tregs exert their immunosuppressive function by expressing high levels of the IL-2 receptor α-chain (CD25). By competitively consuming IL-2, they inhibit the activation of CD8^+^ T cells, thereby promoting the expression of TOX and the exhaustion of CD8^+^ T cells. This process suppresses both autoimmune responses and anti-tumor immunity.^104^^,^^105^ In certain cancer types, such as pancreatic ductal adenocarcinoma, the binding of TNF-α to tumor necrosis factor receptor-2 (TNFR2) on the surface of Tregs further enhances their inhibitory effects, promoting T cell exhaustion.^106^ Additionally, in cases of liver cancer, high expression of the α2 subunit of AMPK, known as PRKAA2, has been found to contribute to T cell exhaustion. This may represent a potential mechanism for immune evasion by cancer cells. The synergistic interaction between T cells and malignant cells with high PRKAA2 expression activates Tregs, further promoting T cell exhaustion.^107^

It is worth noting that in chronic immune activation environments, Tregs themselves may also undergo exhaustion.^85^^,^^108^ Similar to conventional T cells, exhausted Tregs significantly up-regulate the expression of inhibitory receptors (such as PD-1 and TIM3) and transcription factors (including TOX and BLIMP1). Comparable changes also occur in their metabolic pathways and epigenetic landscapes. When Tregs become exhausted, their immunosuppressive function may be weakened or lost, leading to the impairment of immune tolerance mechanisms. This, in turn, may exacerbate transplant rejection reactions.

### Other regulatory cells

Besides Tregs, other types of regulatory cells may also directly or indirectly contribute to T cell exhaustion.^1^ Natural killer (NK) cells possess immunomodulatory functions. Studies have indicated that NK cells can attack CD8^+^ T cells through their surface NKp46 receptors. During the early phase of a high-dose LCMV infection, increased NK cell activity, accompanied by up-regulated NKp46 expression, ultimately leads to NK cell-dependent CD8^+^ T cell exhaustion.^109^ Additionally, NK cells can produce the immunosuppressive cytokine IL-10, which is involved in the process of CD8^+^ T cell exhaustion.

MDSCs possess potent immunosuppressive functions, significantly inhibiting the anti-tumor immune response mediated by CD8^+^ T cells.^110^ MDSCs can be divided into at least two major subpopulations: monocytic MDSCs and polymorphonuclear MDSCs, with monocytic MDSCs exhibiting stronger immunosuppressive capabilities.^116^ Recent studies have indicated that MDSCs can induce CD8^+^ T cell exhaustion. In cases of myelodysplastic syndromes, MDSCs highly express Galectin-9 (Gal9), which binds to the inhibitory receptor TIM-3 on the surface of CD8^+^ T cells, inducing their exhaustion. The mechanism behind this process may involve the AKT/mTOR signaling pathway.^111^ In solid organ transplantation, MDSCs primarily contribute to graft tolerance by suppressing effector T cells.^112^ In contrast, their role in hematopoietic stem cell transplantation is more complex: beyond inhibiting T cell activation and proliferation to prevent GVHD, the expansion of MDSCs post-transplantation may also be associated with tumor relapse.^113^

Macrophages can be mainly divided into two distinct subpopulations, namely M1 and M2. Among them, M2 macrophages primarily produce anti-inflammatory cytokines, such as IL-10 and TGF-β.^114^ Studies have indicated that M2-type tumor-associated macrophages express ligands for various CD8^+^ T cell inhibitory receptors, including PD-L1, CTLA-4 ligands, TIGIT ligands, and TIM-3 ligands, which are associated with terminally exhausted CD8^+^ T cells. Additionally, these exhausted CD8^+^ T cells are capable of secreting factors (such as CSF1 and MIF), which promote the polarization of macrophages towards the M2 phenotype.^115^

### Cytokines

TGF-β stands as a pivotal cytokine in inducing T cell exhaustion, exerting its effects through multiple pathways. On one hand, exhausted T cells display up-regulated expression of transcription factors, nuclear receptor subfamily 4 group A member 2 (NR4A2) and Maf, a process in which TGF-β plays an inductive role. For instance, the synergistic action of TGF-β and IL-6 can significantly elevate Maf expression levels.^116^ On the other hand, TGF-β also suppresses the mTOR signaling pathway within the CD8^+^ exhausted T cell subpopulation, serving as a critical determinant of the precursor metabolism and function of exhausted T cells. These precursors play a vital role in maintaining the homeostasis and self-renewal of exhausted T cells, thereby driving the progression of T cell exhaustion.^117^ It is important to note that TGF-β can also inhibit T cell function through other means, rather than solely inducing exhaustion. For example, in the tumor environment, TGF-β inhibits the activation of anti-tumor T cells by up-regulating the expression of the Foxp1 transcription factor, thereby promoting immune evasion of the tumor.^118^ In the context of chronic viral infections, TGF-β mediates the apoptosis of virus-specific CD8^+^ T cells through intracellular signaling pathways, leading to persistent viral infection.^14^

IL-10 plays an immunomodulatory role in various physiological processes. In chronic viral infections, IL-10 can promote the process of CD8^+^ T cell exhaustion, thereby impeding virus clearance.^119^ IL-10 suppresses the function of antigen-presenting cells, leading to reduced T cell activation and proliferation or contributing to the onset of exhaustion. Furthermore, IL-10 can directly inhibit the effector functions of CD8 and CD4 T cells, including the production of inflammatory cytokines such as IFN-γ and TNF-α, as well as cytotoxicity. This acceleration of T cell functional decline ultimately results in T cell exhaustion. Additionally, studies have shown that blocking the IL-10R signaling pathway reduces the expression level of PD-1 in virus-specific T cells, suggesting that IL-10 may synergize with other negative regulatory pathways, such as PD-1/PD-L1, during the exhaustion process.^120^ In certain tumor models, such as breast cancer and melanoma, IL-10 has been found to promote T cell exhaustion.^121^^,^^122^ Conversely, in human colon cancer models, blocking IL-10 enhances anti-tumor immune function.^123^ However, in some other tumor models, IL-10 maintains the body’s immune surveillance function.^124^ Moreover, research has revealed that IL-10 can directly act on terminally exhausted CD8^+^ T cells, restoring their proliferative capacity and cytotoxicity through metabolic reprogramming. Therefore, the “paradoxical” functions exhibited by IL-10 in immune regulation warrant further investigation.

Several additional cytokines can also contribute to T cell exhaustion. For instance, in chronic infections and tumor microenvironments, IL-6 can promote T cell exhaustion through the IL-6/signal transducer and activator of transcription 3 (STAT3)/PD-1 transcriptional regulatory axis, and may also indirectly influence exhaustion by modulating TGF-β-induced differentiation of Tregs.^125^ Excessive production of IFN-γ may activate the JAK/STAT signaling pathway, induce downstream nitric oxide production, and trigger apoptosis, thereby further driving T cell exhaustion.^126^

Certain signaling pathways, such as those mediated by forkhead box P3 (FOXP3) and IL-2Rα, play critical roles in the function of Tregs and the activity of cytokines like TGF-β. IL-2Rα (CD25) serves as the alpha chain of the IL-2 receptor. Binding of IL-2 to IL-2Rα activates downstream signaling pathways that promote the expression of FOXP3 and enhance Treg stability.^127^ FOXP3, a master transcription factor defining Treg identity, is regulated by both TGF-β and IL-2 signaling. It not only drives the expression of immunosuppressive genes characteristic of Treg (*e.g.*, Tnfrs9) but also represses pro-inflammatory genes like Ptpn22, thereby contributing to immune suppression and the maintenance of immune homeostasis and tolerance.^128^

### Clinical challenges and future directions

Although no clinical trials have yet clearly defined the “optimal” level of T cell exhaustion post-transplantation, several methods are available to monitor its extent to balance immune tolerance against infection risk. Flow cytometry can be used to detect surface exhaustion markers, such as PD-1, Tim-3, and CTLA-4, on T cells in peripheral blood. For example, elevated PD-1 expression on CD4^+^ effector T cells in liver transplant recipients may indicate T cell exhaustion.^129^ Flow cytometry also enables quantification of CD4^+^CD25^+^Foxp3^+^ Tregs, and the ratio of Tregs to CD8^+^ T cells may reflect the state of immune tolerance.^130^ Functional assessment of T cells can be performed by measuring cytokine secretion (*e.g.*, IFN-γ, IL-2). Furthermore, quantitative reverse transcription PCR allows monitoring of exhaustion-associated gene expression levels, such as that of TCAIM (T cell activation inhibitor, mitochondrial),^131^ providing additional insight into the degree of T cell exhaustion.

Strategies for inducing T cell exhaustion have been discussed in preceding sections. However, given that transplantation serves as a therapeutic intervention for certain cancers (*e.g.*, liver transplantation for hepatocellular carcinoma), a major clinical challenge lies in achieving balanced immunosuppression to reduce rejection risk while preserving anti-tumor immunity. Several pharmacological agents have recently shown promise in addressing this complex therapeutic dilemma.

Immune checkpoint inhibitors function by blocking the interaction between immune checkpoint proteins and their ligands, thereby reversing T cell suppression and restoring T cell-mediated anti-tumor activity. While immune checkpoint inhibitors have demonstrated efficacy in treating recurrent hepatocellular carcinoma after liver transplantation, their use is associated with a significant risk of graft rejection. For instance, early administration of immune checkpoint inhibitors, such as anti-CTLA-4 antibodies, in liver transplant recipients has been linked to severe acute rejection episodes.^132^

Before the administration of immune checkpoint inhibitors, combination therapy with multiple immunosuppressive agents may provide broader suppression of immune responses and reduce the risk of rejection. For example, concurrent use of a calcineurin inhibitor (*e.g.*, tacrolimus) and an mTOR inhibitor (*e.g.*, sirolimus) can more effectively inhibit T cell activation and proliferation.^133^ mTOR inhibitors such as sirolimus and everolimus have been utilized in the context of hepatocellular carcinoma in liver transplant recipients. A meta-analysis of four randomized trials indicated that mTOR inhibitor therapy may reduce the risk of *de novo* or recurrent malignancy (relative risk 0.60, *P* = 0.06), while also significantly lowering the incidence of post-transplant rejection compared with standard regimens (*P* < 0.05).^134^

Calcineurin inhibitors, such as cyclosporine and tacrolimus, are widely used to prevent acute GVHD following transplantation. A study in a murine model of allogeneic hematopoietic stem cell transplantation demonstrated that calcineurin inhibitors inhibit the terminal exhaustion of donor T cells, maintaining most of them in a “transitional” state co-expressing inhibitory receptors and effector molecules. These transitional exhausted T cells can regain anti-leukemic activity upon anti-PD-1 blockade, thereby potentially suppressing post-transplant leukemia relapse.^100^

Metformin, a first-line medication for type 2 diabetes, is effective in controlling hyperglycemia induced by immunosuppressive agents, such as tacrolimus and sirolimus, in renal transplant recipients.^135^ In experimental lung transplant models, metformin has demonstrated anti-fibrotic and immunomodulatory properties, reducing T cell infiltration into allografts and suppressing the proliferation and function of pro-inflammatory CD4^+^ T cells.^136^

In recent years, organ-specific delivery systems have demonstrated considerable potential in post-transplant immunotherapy. Advances in materials science have enabled the development of nanoparticles whose surfaces can be conjugated with various small-molecule ligands, antibodies, or peptides to achieve targeted delivery, while their cores can be loaded with “cargo” such as immunosuppressive drugs.^136^ Through rational design, such nanoparticles can simultaneously modulate multiple pathological processes post-transplantation. For example, a novel nano-inhibitor was engineered by surface-functionalizing nanoparticles with an IL-21R monoclonal antibody and encapsulating tacrolimus within the core. This system enabled precise targeting of T follicular helper cells and effectively suppressed the activation of both T and B cells in co-culture assays. *In vivo* experiments further demonstrated that, compared with conventional tacrolimus, this targeted nano-inhibitor reduced lymphocytic infiltration, lowered inflammatory markers, and diminished nephrotoxicity.^137^

## Conclusion and discussion

T cell exhaustion represents a dysfunctional state of T cells under persistent antigenic stimulation, characterized by the overexpression of checkpoint molecules such as PD-1, CTLA-4, TIM-3, and LAG-3, leading to the attenuation of T cell effector functions. Additionally, T cell exhaustion is accompanied by epigenetic remodeling and metabolic alterations. Epigenetic modifications, including DNA methylation and histone modifications, stably maintain the exhaustion phenotype. Metabolically, progenitor exhausted T cells rely on fatty acid oxidation, while terminally exhausted T cells exhibit reduced glycolytic capacity, concomitant with the accumulation of reactive oxygen species and decreased mTORC1 activity.

In the field of transplantation, T cell exhaustion contributes to graft tolerance but may also weaken immune defense due to excessive exhaustion, thereby increasing the risk of infections and tumorigenesis. Furthermore, the role of T cell exhaustion varies among different organs; for instance, an increase in T cell exhaustion is associated with graft survival in liver transplantation, whereas excessive exhaustion may elevate the risk of infections. The induction mechanisms of T cell exhaustion are diverse, involving the synergistic action of cells such as Tregs, MDSCs, and M2 macrophages. Cytokines like IL-10 and TGF-β also accelerate the exhaustion process by inhibiting effector functions and metabolic reprogramming. Current therapeutic strategies, including metabolic modulation (*e.g.*, metformin) and immune checkpoint blockade, aim to regulate T cell exhaustion and optimize transplant immunology.

Substantial mechanistic insights into T cell exhaustion initially emerged from murine models of chronic LCMV infection and allogeneic transplantation, which established the foundational understanding of T cell exhaustion molecular characteristics and regulatory pathways.^54^^,^^138^ On the clinical front, these findings have been progressively validated: in liver transplantation, studies suggest that the presence of exhausted/immunoregulatory signatures, such as immune checkpoint molecule expression, in the peripheral blood of partially tolerant recipients may correlate with graft acceptance^139^; in kidney transplantation, an increased proportion of PD-1^+^CD57^-^ exhausted T cells after the induction phase has been reported to associate with improved graft function^84^; and in allogeneic hematopoietic stem cell transplantation, donor T cell exhaustion has been linked to a reduced risk of GVHD, though often at the expense of attenuated GVL effects.^100^ These observations indicate that core T cell exhaustion mechanisms are conserved across species, and insights from mouse models have been successfully translated to human transplantation. Nevertheless, current evidence also highlights the organ-specific dual roles of T cell exhaustion across different transplant settings, underscoring the need for further research to enable precise clinical modulation.

In summary, T cell exhaustion not only represents an immune adaptive response under chronic immune stimulation but also plays a significant role in transplantation, cancer immune evasion, and other diseases. Further investigation into its molecular mechanisms and regulatory pathways will provide novel approaches for transplantation immunology, immunotherapy, and the treatment of related diseases. Balancing the benefits of T cell exhaustion with the impairment of immune defense will be key to enhancing the success of transplant immunology and the effectiveness of immunotherapy.

## CRediT authorship contribution statement

**Yining Wang:** Writing – original draft, Visualization, Methodology, Investigation. **You Wu:** Writing – original draft, Methodology. **Yufei Shen:** Visualization, Methodology. **Yujia Chen:** Writing – original draft, Visualization. **Yifan Zhao:** Visualization. **Xiandong Zeng:** Writing – review & editing. **Kang He:** Writing – review & editing, Supervision, Conceptualization.

## Funding

This study is funded by Shanghai Natural Science Foundation (No. 23ZR1438600), Shanghai Research Center for Organ Transplantation, Category A Key Research Center of Shanghai Municipality (No. 2022ZZ01016), Dynamic Evolution and Regulatory Mechanisms of Host Immune Response Before and After Liver Transplantation (Joint Application A), Shanghai Jiao Tong University School of Medicine Affiliated Renji Hospital Technology Achievement Transformation Cultivation Project (No. RJZH26-005), Shanghai Jiao Tong University School of Medicine Science Popularization Cradle Program (No. YL250209), Undergraduate Innovation Training Program of Shanghai Jiao Tong University School of Medicine (No. 20260006), Shanghai Jiao Tong University School of Medicine “Young Science and Technology Innovation Workshop” (GanWeiRenXian), and Special Program of the National Natural Science Foundation of China (No. 82241221).

## Conflict of interests

The authors declared no competing interests.
